# The Activation by Glucose of Liver Membrane Nitric Oxide Synthase in the Synthesis and Translocation of Glucose transporter-4 in the Production of Insulin in the Mice Hepatocytes

**DOI:** 10.1371/journal.pone.0081935

**Published:** 2013-12-03

**Authors:** Suman Bhattacharya, Rajeshwary Ghosh, Smarajit Maiti, Gausal Azam Khan, Asru K. Sinha

**Affiliations:** 1 Department of Biochemistry, Sinha Institute of Medical Science and Technology, Kolkata, India; 2 Department of Biochemistry, Vidyasagar University, Midnapore, India; Saint Louis University, United States of America

## Abstract

**Introduction:**

Glucose has been reported to have an essential role in the synthesis and secretion of insulin in hepatocytes. As the efflux of glucose is facilitated from the liver cells into the circulation, the mechanism of transportation of glucose into the hepatocytes for the synthesis of insulin was investigated.

**Methods:**

Grated liver suspension (GLS) was prepared by grating intact liver from adult mice by using a grater. Nitric oxide (NO) was measured by methemoglobin method. Glucose transporter-4 (Glut-4) was measured by immunoblot technique using Glut-4 antibody.

**Results:**

Incubation of GLS with different amounts of glucose resulted in the uptake of glucose by the suspension with increased NO synthesis due to the stimulation of a glucose activated nitric oxide synthase that was present in the liver membrane. The inhibition of glucose induced NO synthesis resulted in the inhibition of glucose uptake. Glucose at 0.02M that maximally increased NO synthesis in the hepatocytes led to the translocation and increased synthesis of Glut-4 by 3.3 fold over the control that was inhibited by the inhibition of NO synthesis. The glucose induced NO synthesis was also found to result in the synthesis of insulin, in the presence of glucose due to the expression of both proinsulin genes I and II in the liver cells.

**Conclusion:**

It was concluded that glucose itself facilitated its own transportation in the liver cells both via Glut-4 and by the synthesis of NO which had an essential role for insulin synthesis in the presence of glucose in these cells.

## Introduction

Glucose has been reported to have a critically important role both in the synthesis and in the secretion of insulin in the islets of Langerhans [1,2] which is currently believed to be the only site of production and secretion of the hypoglycaemic protein. We, on the other hand, have recently reported that a glucose dependent synthesis and secretion of insulin also occurred in the hepatocytes in adult mice [3]. It should be mentioned here that various etiological and demographical studies have previously suggested that the liver could have a significant contribution in both the long and short term of glucose homeostasis [4], and the chronic hepatitis is reported to lead to diabetes mellitus [5]. It has also been reported that rat hepatic stem cells cultured in high glucose concentration were capable of producing insulin and the hypoglycaemic hormone was ubiquitously present in extra pancreatic tissues of rat and humans [6,7]. Although insulin is essential for the synthesis of hepatic glycogen synthesis, as much as 50% of the pancreatic insulin was reported to be destroyed in the liver suggesting that liver itself might have synthesized insulin essential for the glycogen synthesis. 

It is also known that although insulin receptors are present on hepatic cells [8], the uptake of glucose by the hepatic cells has been reported to be independent of insulin [9]. The mechanism of glucose uptake in the hepatic cells, an insulin independent process, remains obscure. A glucose transporter protein (MW 54Kda) known as glucose transporter- 4 (Glut-4) is reported to play a critically important role in the importation of the sugar into the hepatic cells from the external milieu through its translocation in the cell membrane both in the insulin dependent [10] and insulin independent processes [11]. Although the availability of Glut-4 is considered to be critically important for the maintenance of the glucose homeostasis, no report on the mechanism of the synthesis of this transporter protein itself is available. In the context that glucose was capable of stimulating the synthesis and secretion of insulin in the liver cells [3], similar to that in the pancreatic β cells [12], the influx of glucose from the circulation into the hepatic cells presented a potentially difficult problem particularly because of the presence of Glut-2 in the liver cells that favours the efflux of glucose from the liver cells into the circulation for the maintenance of systemic glucose homeostasis to counteract the development of hypoglycaemia [13]. Moreover the transportation of glucose in the liver cells as reported above was an insulin independent process [14], and, as such, the role of insulin itself for the translocation of Glut-4 to facilitate the influx of glucose into the liver cells in the presence of the opposing effect of Glut-2 in the hepatocytes remains obscure. Investigation was carried out to find the role of glucose itself, if any, on the transportation of the sugar into the liver cells through the synthesis of NO. Particularly, because the oxide had been reported before to facilitate the glucose transport into the pancreatic β cells [12].

We report herein the existence of a novel constitutive form of nitric oxide synthase (cNOS) in the mice liver cell membrane that was found to be stimulated by glucose leading to the synthesis of NO in the mice hepatocytes. The role of glucose induced NO production both in the liver membrane translocation of Glut-4 as well as in the synthesis of Glut-4 in the hepatic cells for the expression of the genetic elements leading to the synthesis of insulin by the sugar in the liver were investigated [3]. 

## Materials and Methods

### Ethics Statement

The protocol used in this study was approved by the Internal Review Board, Sinha Institute of Medical Science and Technology, Kolkata. The required approval was obtained for the use of animals in the study by the Internal Review Board for Animal Care, Sinha Institute of Medical Science and Technology, Kolkata, consisting of a special committee for animal care and their use that oversaw the welfare, care and nutritional requirements for all the animals used in the study. The committee had a permanent certified veterinarian whose duty is to ensure that the all the animals were free from any diseases as stipulated by the Animal Right Group. All animal related experiments were strictly performed in the presence of a member of the Animal Right Group and under the supervision of the veterinarian, and special care were taken to ensure that no animals were unnecessarily harmed or were subjected to pain during the study. After the termination of the study, the animals were sacrificed by euthanasia in a carbon dioxide chamber.

White albino healthy mice (20-25gm each), Swiss strain, irrespective of gender were used for the study [15]. These inbred animals were fed standard laboratory chow and sterilized water was given *ad libitum*. The animals were kept under 12h cycles of light and dark at 23°C.

### Chemicals

The Goat anti-rabbit immunoglobulin G-alkaline phosphatase, HEPES (4-(2-hydroxyethyl)-1-piperazineethanesulfonic acid) were obtained from Sigma Aldrich co, Glut-4 antibody was obtained from Santa Cruz Biotech. Insulin antibody was also obtained from Santa Cruz Biotech (Insulin H-86). ELISA Maxisorb plates were from Nunc, Roskilde, Denmark. All other chemicals were of analytical grade.

### Preparation of mice liver membrane

Typically, adult mice were killed by cervical dislocation. The liver membrane was prepared by gently cutting the whole liver (2.40gm) into small pieces that were collected in HEPES buffer (pH 7.4) without glucose at 0°C and immediately homogenized by freezing and thawing by using liquid N_2_ for 5-6 times. The homogenized mass was next centrifuged at 60,000g for 60 min at 0°C. The supernatant fraction was discarded and the pelleted fraction that contained the membranes were suspended in HEPES buffer (pH 7.4) and used for further studies as soon as possible.

### Stimulation of glucose activated nitric oxide synthase (GANOS) in mice liver membrane preparation

The mice liver membrane in HEPES buffer (pH 7.4), was prepared as described above. Typically, 1-2mg of the crude membrane preparation was incubated with different amounts of glucose as described in the presence of 2mM CaCl_2_ in a total volume of 1.0ml. After incubation at 37°C for 30 min, the amount of NO formed in the reaction mixture was determined by the conversion of oxyhemoglobin to methemoglobin by the spectral changes of absorption maxima at 525 and 630 nm under N_2_ as described [16]. The quantitation of NO was independently verified by chemiluminescence method [17]. In some of the experiments identical reaction mixtures were treated with different concentrations (1.0 mM to 0.1 mM) of NAME (N^G^-methyl-l-arginine acetate ester), an inhibitor of nitric oxide synthase [18], and the formation of NO was similarly determined.

### Construction of Lineweaver-Burk plot of GANOS

 Typically, the reaction mixture containing 0.1mg of the crude membrane preparation was incubated with 2mM CaCl_2_ and different concentrations of *l-*arginine and 0.02M glucose at 37°C for 30 min, the amounts of NO formed in the reaction mixture was determined as described above. In a parallel experiment identical reaction mixture containing *l-*arginine but without the added glucose was incubated at 37°C for 30 min and the formation of NO determined and Lineweaver-Burk plot was similarly constructed.

### Preparation of grated liver suspension (GLS) from the mice liver

 The grated liver suspension (GLS) was prepared by gently rubbing the whole liver from adult mice against a cold (0°C) stainless steel wire grater (average grater size 3mm) that yielded ≈ 8mg chunks of the liver. Subsequently the chunks were cut by microtome which were immediately collected in HEPES buffer (pH 7.4) without glucose at 0°C, and used for further studies as soon as possible. As described elsewhere in this manuscript, the hepatic cells within these “chunks” were found to retain their structural integrity with minimal damage. GLS thus prepared was morphologically verified and was confirmed to contain hepatocytes by microscope. GLS was dissected using microtome and observed under microscope which showed distinct hepatic cells characteristics. 

### Determination of glucose uptake by the mice GLS

The rate of glucose uptake by the GLS was determined by using non metabolizable 2-deoxy-D-^14^C glucose as described [19]. 

### Immunohistochemical localization of Glut-4 in the mice hepatocytes

The mice liver “chunks” were sliced into 6-8 nm sections in a cryostat to demonstrate the presence of Glut-4 in the hepatocytes. These sections were incubated with Glut-4 antibody (1:200 dilutions) and identified by using fluorescent tagged anti-rabbit immunoglobulin G-alkaline phosphatase as described [20]. Following the immunohistochemistry, sections were imaged by using a fluorescent microscope attached to a high resolution digital colour camera which photographically recorded the non-weighted images of the sections.

### Identification of Glut-4 by immunoblot and its quantitation by enzyme linked immunosorbent assay (ELISA) in the supernatant of the glucose treated disrupted GLS

The amounts of Glut-4 synthesized in the crude supernatants of GLS incubated with different concentrations of glucose (0 to 0.05M) were identified by immunoblot [21] and quantitated by ELISA [22] by using Glut-4 antibody. The Enzyme linked immunosorbent assay was performed as described [22]. Briefly, Glut-4 was incubated with an equal volume of phosphate buffer saline (PBS) in an assay plate overnight in 4°C. Nonspecific binding was blocked by 5% bovine serum albumin in the same buffer. The samples were then washed with PBS containing Tween-20, and incubated for 2h with diluted primary antibody in PBS (1:200) obtained from Santa Cruz biotech. The samples were next washed with PBS-T20 and incubated with diluted goat antirabbit IgG-alkaline phosphatase (1:2000) in the same buffer for 1h. After washing they were incubated with *p*-nitrophenyl phosphate (1mg/ml) in carbonate buffer (pH 9.8) containing 10mM MgCl_2_ . The development of colour was determined at 405nm. The amount of Glut-4 present in the sample was determined in an ELISA reader. The density of the immunopositive protein bands in the immunoblot were quantitated by using Image J program by computer analysis. Due to the unavailability of pure Glut-4 as the antigen, the quantitation of Glut-4 was carried out by using Image J program and expressed in terms of arbitrary optical density (O.D) only for the Glut-4 immunopositive bands in the gel and compared to the control where no glucose was added to the reaction mixture. The absence of Glut-4 immunopositive band in the SDS gel served as the control as described below. 

### Separation of RNA

The grated liver suspension in HEPES buffer (pH 7.4) (8mg/ml) was incubated with different concentrations of glucose for 30 min. After incubation the nucleic acids which contained Glut-4 mRNA from the sugar treated GLS were extracted using Trizol-chloroform method [23]. To one ml of each cell suspension 0.5 ml of Trizol was added to lyse the cells, later 0.5 ml of chloroform per 1 ml of Trizol was added. The samples were then centrifuged at 12,000 rpm for 15 min at 4°C. Following centrifugation, the mixture separated into a lower red phenol-chloroform phase, an interface, and a colourless upper aqueous phase. Total RNA remained exclusively in the aqueous phase, the aqueous phase was transferred into an eppendroff, to precipitate RNA, and 1 ml isopropanol per 1 ml of Trizol was added. It was then centrifuged at 12,000 rpm for 10 min at 4°C. RNA pellet was then washed with 75% ethanol and then centrifuged at 7500 rpm for 5 min at 4°C. RNA was dissolve in RNAse free water.

### Separation of plant ribosomes

Bael leaf (Aegle marmelos) around 10 gm was taken and washed to remove debris twice and rinsed in distilled water and homogenized and later centrifuged at 5000 rpm to remove the debris, the supernatant was taken, and centrifuged at 13,000 x g. The supernatant was layered on top of a 1mM sucrose cushion and centrifuged at 200,000 x g to pellet ribosomes as described [24].

### In vito translation of Glut-4 mRNA

RNA was isolated by Trizol-chloroform method from GLS described above [23]. Briefly the mRNAs were incubated with ribosomal preparation, mixture of all 20 amino acids (0.1µmol/ml, each) and 2mM ATP as described [25]. After 6 h the reaction mixture was centrifuged at 10,000 g at 0°C for 10 min. The supernatant was used for the determination of Glut-4 by ELISA as described above. 

### Immunoblot analysis of Glut-4 in the GLS of mice hepatocytes

Typically, the pelleted fraction from the disrupted GLS as described above was collected. The presence of Glut-4 in the pelleted fraction of GLS of mice hepatocytes treated with different amounts of glucose and incubated for 30 min at 37°C. The presence of Glut-4 in the fraction was identified by immunoblot technique. The samples were subjected to SDS-polyacrylamide gel electrophoresis and stained with Coomassie brilliant blue [26]. An identical SDS gel that was not stained with Coomassie brilliant blue also prepared. The protein bands were next transfer to a nitrocellulose membrane [21], and Glut-4 was subsequently identified in the nitrocellulose membrane by using fluorescent tagged Glut-4 antibody (1:200 dilutions). 

### Determination of the glucose induced synthesis of insulin

The glucose induced synthesis of insulin in the mice hepatocytes due to the expression of both proinsulin genes I and II in mice [3] was determined by incubating the GLS in HEPES buffer (pH 7.4) with different concentrations of glucose for 30 min at 37°C. After incubation the extracted nucleic acids which contained insulin-mRNA was translated *in vitro* by using plant ribosomal particles as described before and synthesized insulin was subsequently quantitated by ELISA [22] by using both anti-insulin antibody and commercial insulin antibody. The synthesis of insulin in the assay mixture was confirmed by bioassay of the hormone as described before [3].

 The expression of both proinsulin genes I and II were determined by cDNA preparation as described before [3].

### Statistical Methods

 The results shown are mean ± standard deviation (SD) of at least 5 experiments using 5 different animals, each carried out in triplicate. The significance of the results was analyzed by Student’s t test. Significance (p<0.05) was considered to be significant.

## Results

### Effect of glucose on the activation of nitric oxide synthase in liver cell membrane from adult mice

**Figure 1:**
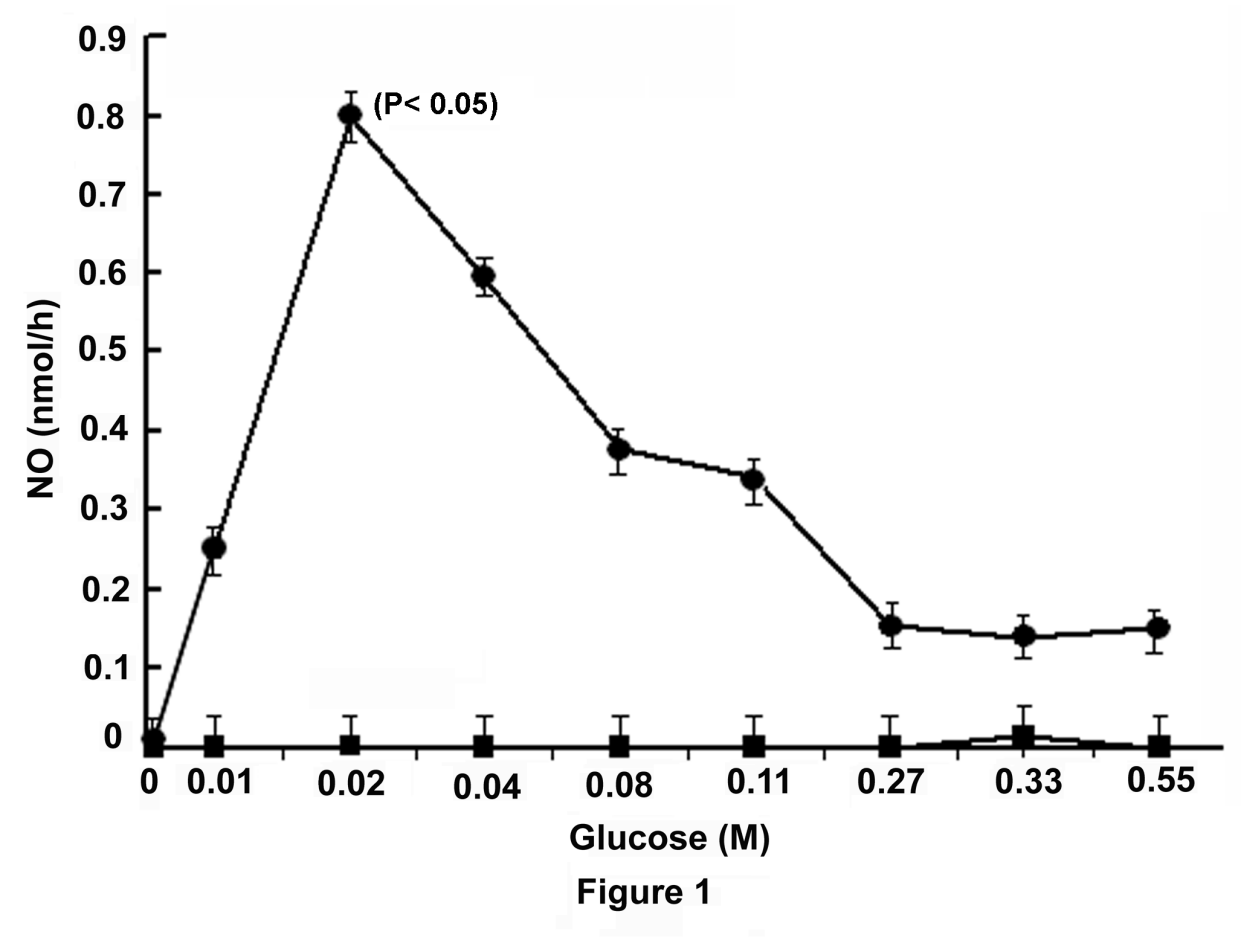
Stimulation of NO synthesis by glucose in mice liver membrane and its inhibition by NAME. The liver membrane in HEPES buffer, pH 7.4, was prepared from the liver of adult mice as described in the Materials and Methods and incubated with different amounts of glucose in the presence of 2.0mM CaCl_2_ in a total volume of 1.0ml for 30 min at 37°C. The synthesis of NO was determined by methemoglobin method as described in the Materials and Methods. Identical reaction mixtures contained 0.1mM NAME were incubated under identical conditions and the synthesis of NO was similarly determined. Each point represents mean ± S.D. of 5 experiments by using 5 different animals each in triplicate. The solid circles (●) represent the synthesis of NO in the liver membrane preparation in the presence of glucose alone, the solid squares (■) represent the synthesis of NO in the liver membrane preparation in the presence of both glucose and NAME.

As the treatment of islets of Langerhans with glucose has been reported to result in the stimulation of NO synthesis [12], to determine the possibility of the occurrence of the glucose induced synthesis of NO in the liver cells, the liver membrane preparation was treated with different amounts of glucose to determine the possible occurrence of the glucose activated nitric oxide synthase (GANOS) in the membrane preparation. The treatment of the liver cell membrane preparation with different amounts of glucose as indicated (Figure 1) was found to result in the increased synthesis of NO, and at 0.02M glucose the liver membrane GANOS activity was found to be maximally stimulated. Addition of 0.1mM NAME, an inhibitor of NO synthesis [18], was found to completely nullify the stimulatory effect of the added glucose on GANOS at all concentrations of glucose used in the synthesis of NO in the assay mixture. (n=15, p< 0.001)

### Lineweaver-Burk plot of the stimulation of GANOS by glucose in the liver membrane preparation

**Figure 2:**
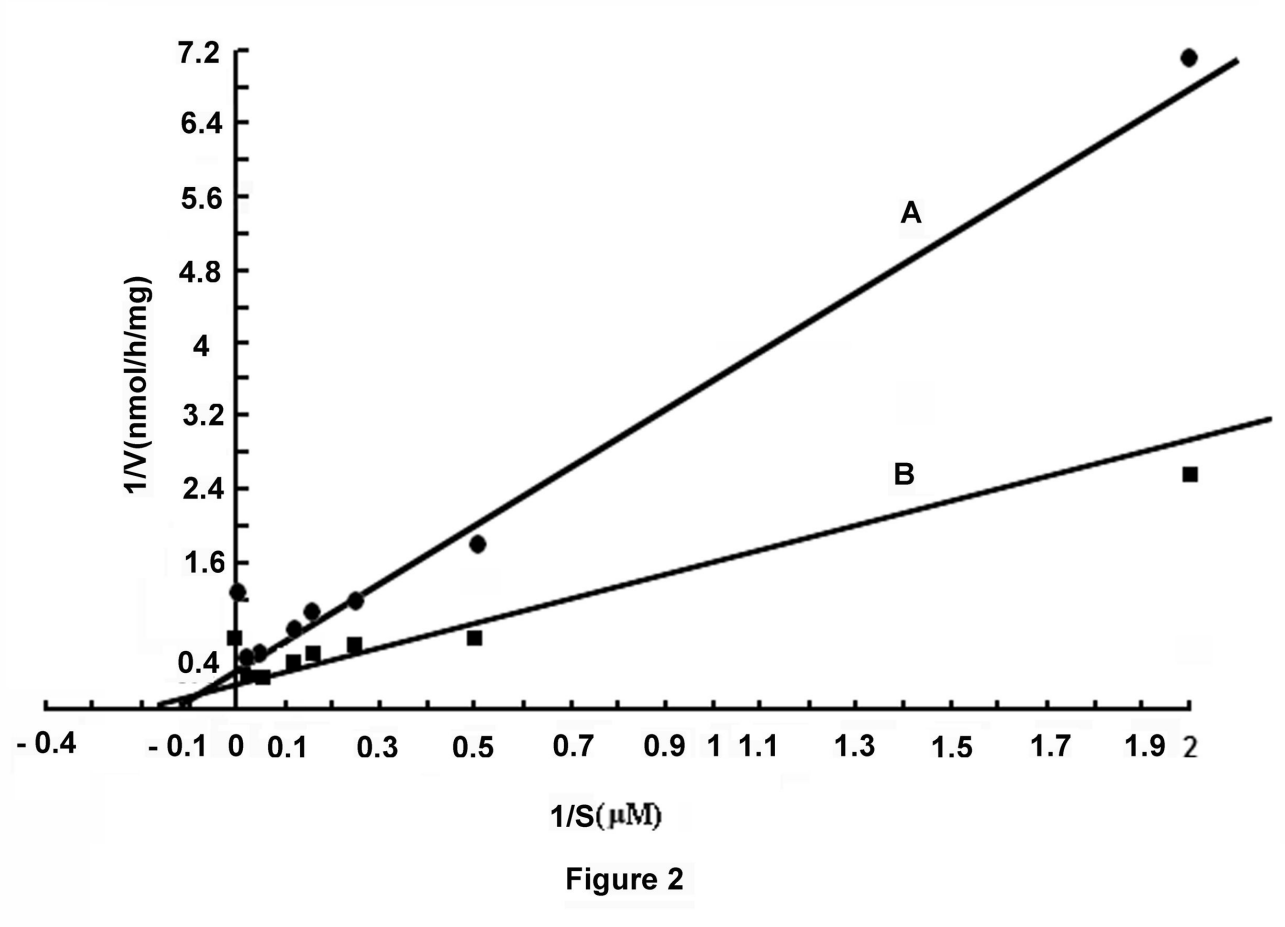
Line-Weaver Burk plot of the stimulation of glucose induced NO synthesis in mice liver preparation. The mice liver membrane in HEPES buffer, pH 7.4, was incubated in the presence and absence of 0.02M glucose with different amounts of *l*- arginine and 2.0mM CaCl_2_ in a total volume of 1.0ml. After incubation for 30 min at 37°C the synthesis of NO was determined by methemoglobin method as described in the Materials and Methods. Line A represents the synthesis of NO in the absence of added glucose to the reaction mixture with different concentrations of *l*-arginine. Line B represents the synthesis of NO in the reaction mixture in the presence of 0.02M glucose and with different amounts of *l*-arginine.

Lineweaver-Burk plot of the activity of GANOS in the liver membrane preparation, in the presence of 0.02M glucose, indicated that the basal GANOS activity (i.e. the basal activity in the absence of the added glucose) [Km= 10µM, Vmax= 2.2nmol NO formed/mg protein/h] of the membrane preparation (Line A, Figure 2) that indicated NO production by GANOS was stimulated due to the addition of 0.02M glucose in the reaction mixture [Km= 5.68µM, Vmax= 3.131nmol NO formed/mg protein/h] (Line B, Figure 2).

### Effect of glucose induced NO synthesis in the GLS in the uptake of the sugar by the suspension

**Figure 3:**
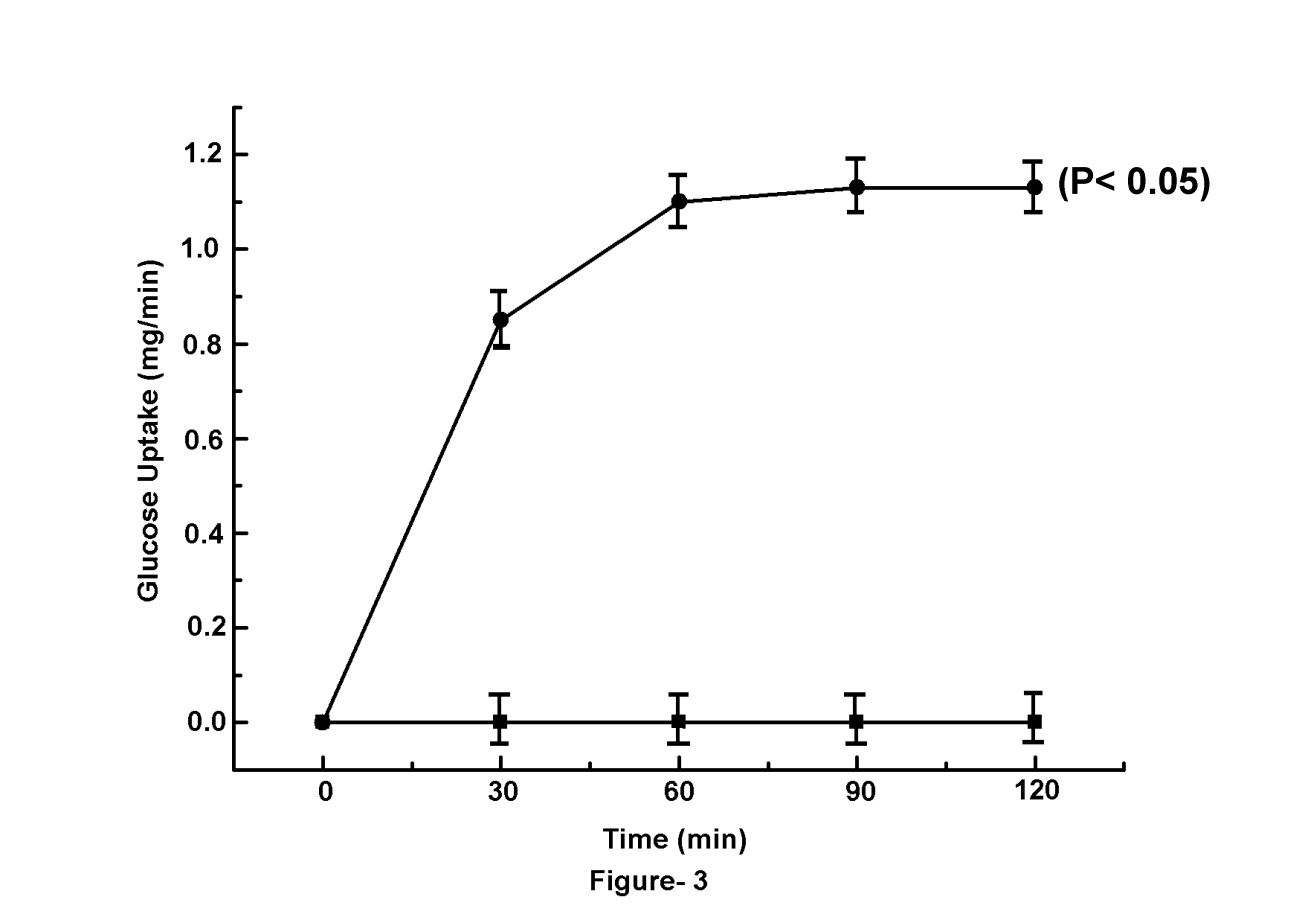
Time course of glucose uptake by the GLS in the presence and absence of NAME. The grated mice liver suspension (GLS) in HEPES buffer pH (7.4), was prepared as described in the Materials and Methods. Typically, 30-40 mg of GLS in the HEPES buffer, pH 7.4, was incubated in the presence of 0.02M nonmetabolizable 2-deoxy-D- glucose with 1µl of the ^14^C-labelled sugar as described in the Materials and Methods for different times as indicated. In parallel experiments, 0.1mM NAME was added to the identical reaction mixture and glucose uptake in both cases was determined. Results are mean ± S.D. of 5 experiments each in triplicate using GLS preparation from 5 different animals. Solid circles (●) represent the uptake of glucose by GLS in the presence of 0.02M glucose and solid squares (■) represent the uptake of glucose by GLS in the presence of both glucose and NAME in the reaction mixture respectively.

 The treatment of GLS with different amounts of glucose not only resulted in the increased synthesis of NO by the suspension as described (Figure 1), but was also found that the increased synthesis of NO resulted in the increased uptake of glucose by the suspension, and at 120 min, the uptake of glucose was maximally (1.1±0.06 mg/min) achieved. The addition of 0.1mM NAME to the reaction mixture that completely inhibited NO synthesis was found to simultaneously abolish the glucose induced glucose transport in the GLS (Figure 3) (n=15, p<0.001)

### The glucose induced translocation of Glut-4 in the liver cells membrane

The results described in Figure 3 demonstrated that the uptake of glucose in the GLS was found to be gradually increased with time, and at equilibrium, the K_equilibrium_ reached to 1.11 indicating that there was an appreciable increase of the glucose uptake at equilibrium, due to NO synthesis induced by the sugar in the GLS (Figure 3)

**Figure 4:**
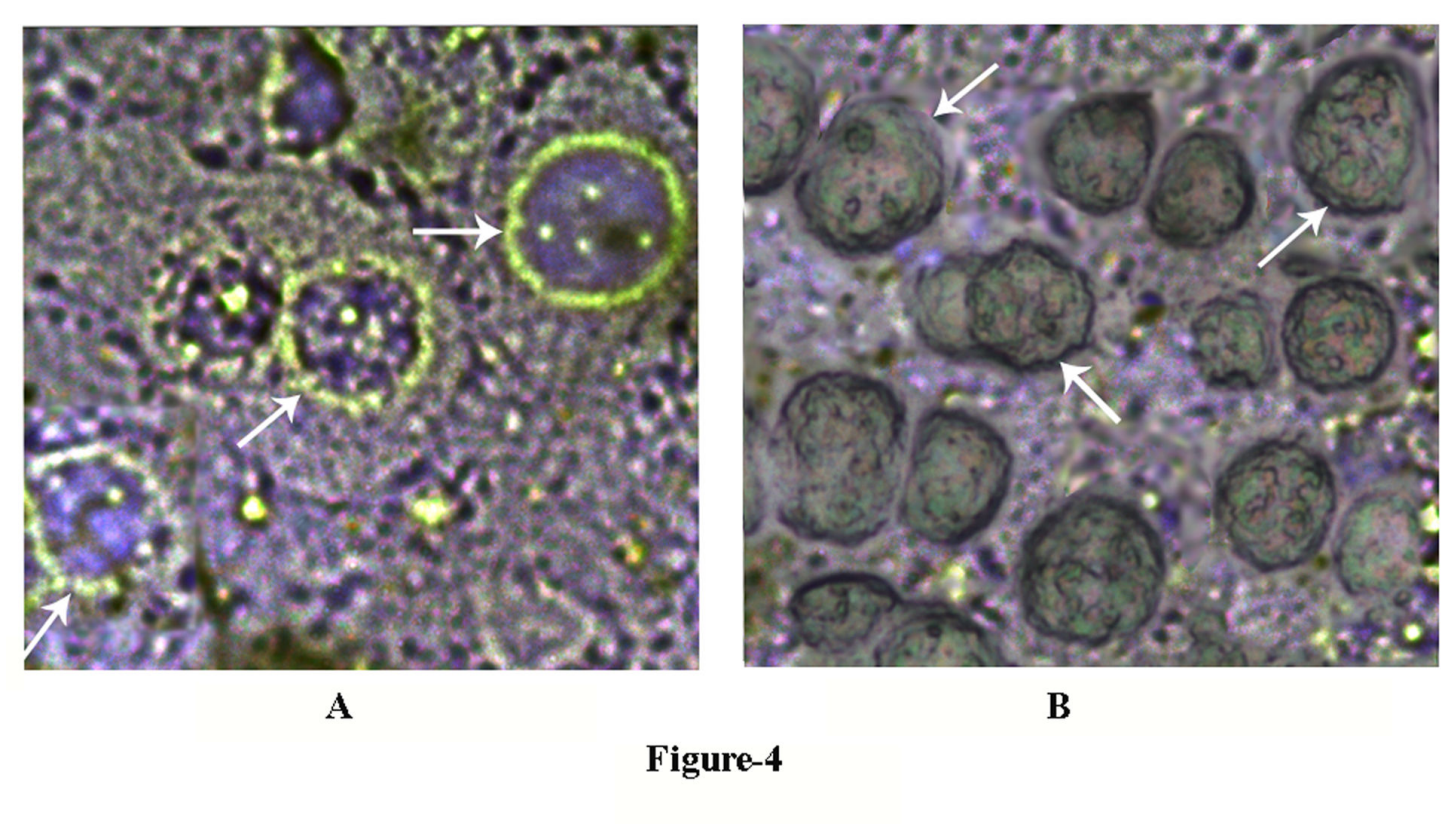
The appearance of Glut-4 on membrane peripheries of hepatocytes incubated with glucose with and without NAME. The grated liver suspension in HEPES buffer, pH 7.4, was incubated with 0.02M glucose in the presence and absence of 0.1mM NAME in the incubation mixture for 30 min at 37°C. After incubation GLS “chunks” were sliced into 6-8 nm sections by using a cryostat. The sliced sections were treated with Glut-4 antibody to demonstrate the presence of Glut-4 by immunohistochemistry as described in the Materials and Methods. Panel-A: The immunohistochemistry of liver sections was incubated in the presence of 0.02M glucose. Glut-4 in the liver cells was determined by using fluorescent tagged Glut-4 antibody. White arrow indicates the translocation of Glut-4 transporter to the periphery of liver cell membrane. Panel-B: The immunohistochemistry of liver sections was incubated in the presence of both 0.02M glucose and 0.1mM NAME by using fluorescent tagged Glut-4 antibody as described in the case of panel-A. White arrow indicates the absence of translocation of Glut-4 transporter to the membrane periphery. The figures shown are typical representative of six different experiments using 6 different animals.

 Experiments were conducted to determine whether the glucose induced NO synthesis in the liver cells (Figure 1) was due to the translocation of Glut-4 in the hepatocytes membrane to facilitate the sugar transport. It was found that the incubation of GLS with glucose resulted in the translocation of Glut-4 molecules in the cell membrane peripheries as visualized by the immunohistochemical microscopy by using fluorescent tagged Glut-4 antibody (Figure 4A). When the same preparation was treated with 0.1mM NAME that inhibited NO synthesis, before the cells were treated with glucose, the presence of Glut-4 in the liver membrane periphery by the immunohistochemical technique could not be demonstrated (Figure 4B). 

### The synthesis of Glut-4 in GLS in the presence of glucose

**Figure 5:**
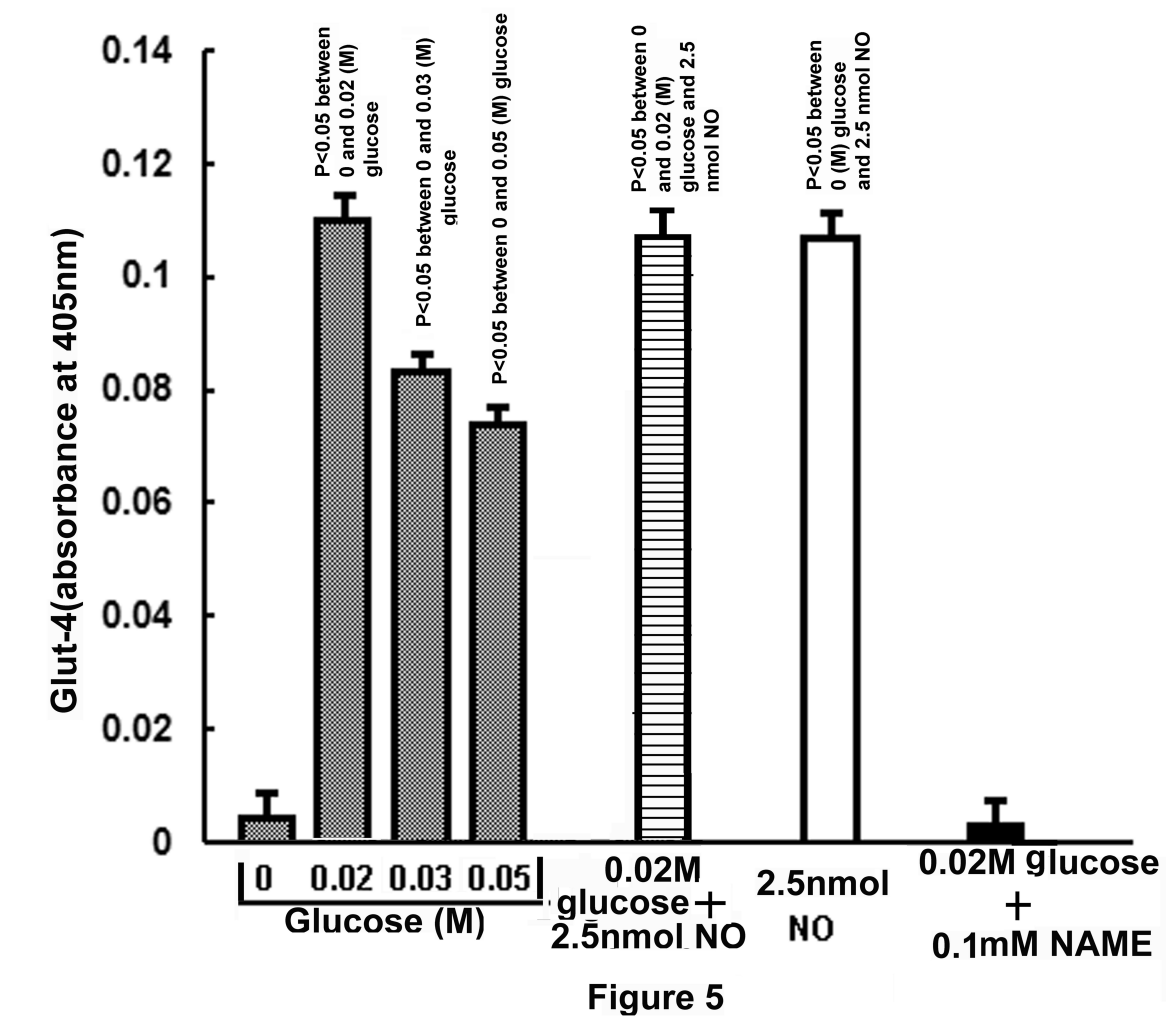
Synthesis of Glut-4 in GLS incubated either with different amounts of glucose or with NO. Typically 30 to 40 mg of GLS in HEPES buffer, pH 7.4, was incubated with different concentrations of glucose (0-0.05M) as shown or in the presence of 2.5nmol NO solution in 0.9% NaCl or in the presence of both 0.02M glucose and 2.5nmol NO solution for 30 min at 37°C. In parallel experiments, 0.1mM NAME was added to the reaction mixture containing 0.02M glucose. After incubation for 30 min at 37°C, the amounts of Glut-4 produced in the reaction mixture were determined by ELISA using Glut-4 antibody. The amount of Glut-4 produced in the reaction mixture was determined by expressing the increase of the arbitrary optical densities at 405 nm. ▓ - Glut-4 synthesis in the presence of varying amounts of glucose; ≡ - Glut-4 synthesis in the presence of both glucose and NO; **□**- synthesis of Glut-4 in the presence of only NO; ■- Glut-4 synthesis in the presence of glucose and NAME. Results shown are mean ± S.D. of the optical densities obtained from 6 experiments by using 6 different animals.

As glucose was found to increase the synthesis of NO in the liver cells (Figure 1), efforts were made to determine the possible effect of glucose induced NO production in the liver cells in the synthesis of Glut-4 itself, a protein. It was serendipitously found that the glucose induced NO production also resulted in the synthesis of Glut-4 in the liver cells as quantitated by ELISA by using Glut-4 antibody (Figure 5).

It was found that the incubation of GLS with different amounts of glucose that resulted in the increased synthesis of Glut-4 was maximally increased at 0.02M glucose in the incubation mixture. Further increase of the sugar concentrations in the reaction mixture, however not only resulted in the decreased synthesis of NO as described in the Figure-1, but was also found to result in the reduced production of Glut-4. The absence of the added glucose in the reaction mixture resulted in the production of Glut-4 which was equivalent to 0.01 arbitrary (O.D) units in the ELISA. The use of 0.02M glucose in the same reaction mixture resulted in the increase of the O.D, due to the increased production of the glucose transporter to 0.12±0.006. In other words, the production of Glut-4 at 0.02M glucose was found to be increased by < 12 times when compared to that synthesized in the absence of the added glucose in the reaction mixture (Figure 5). It was further found that the addition of NAME (0.1mM) with glucose (0.02M) in the reaction mixture resulted in the complete inhibition of glucose induced increased syntheses of both NO and Glut-4 production in the GLS. On the other hand, the addition of 2.5nmol NO solution in 0.9% NaCl instead of glucose itself led to the synthesis of Glut-4 in the liver cells preparation which was similar to that synthesized in the presence of 0.02M glucose in the reaction mixture (Figure 5). The addition of 0.1mM NAME to the reaction mixture containing NO had no effect on Glut-4 synthesis but the addition of NAME to the reaction mixture containing glucose completely inhibited the synthesis of the transporter, indicating NAME caused the inhibition of Glut-4 synthesis stimulated by glucose due to the inhibition of NO synthesis induced by glucose (n=6, p< 0.05). The addition of NAME to the reaction mixture had no effect of the added NO induced Glut-4 synthesis in the reaction mixture indicating NAME itself is not the inhibitor of Glut-4 synthesis.

**Figure 6:**
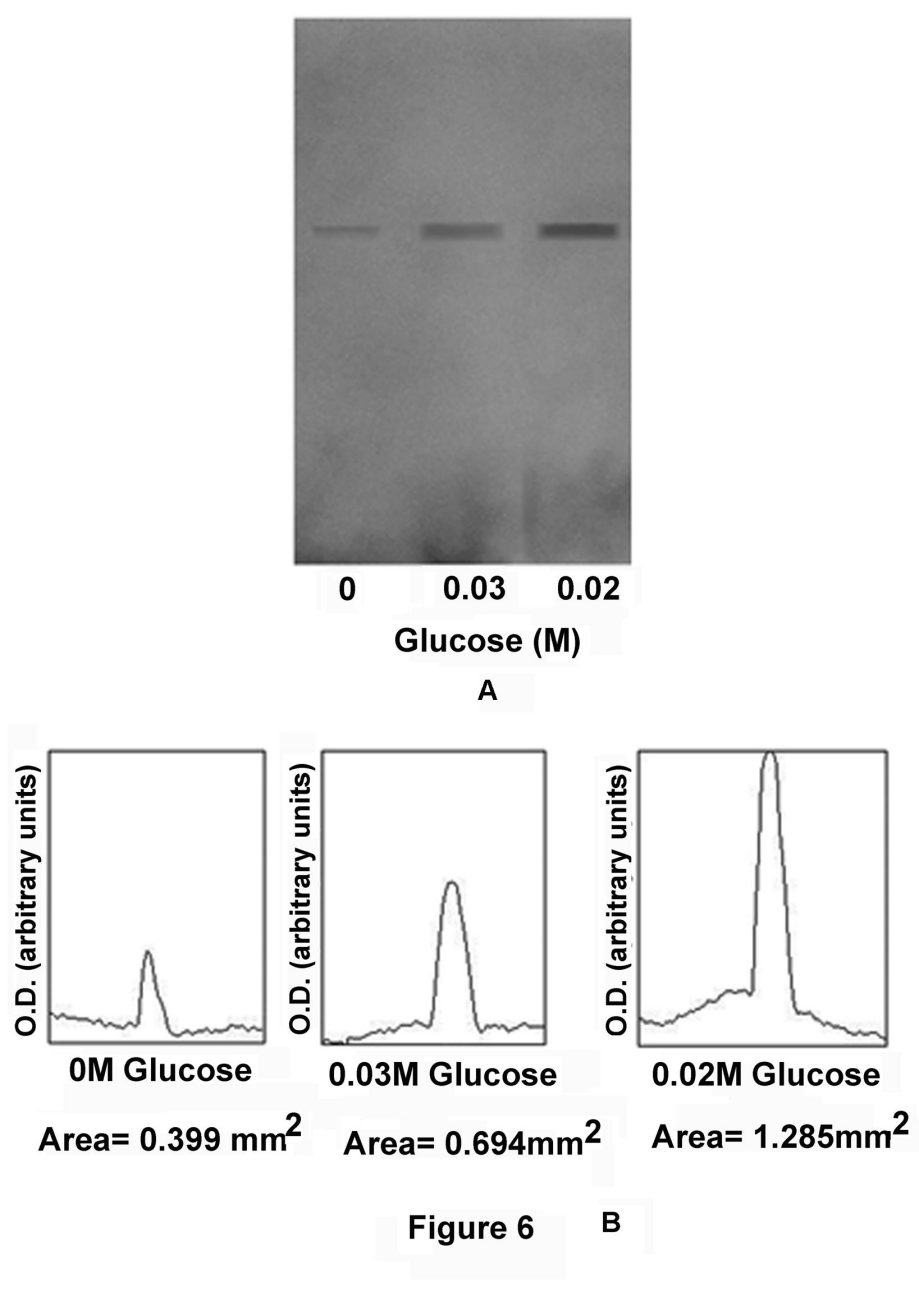
Immunoblot analysis of Glut-4 synthesis by in vitro translation of Glut-4 mRNA in the GLS. The grated liver suspension in HEPES buffer, pH 7.4, was incubated with different concentrations of glucose for 30 min at 37°C. After incubation, nucleic acids which contained mRNA of Glut-4 were extracted and translated *in* *vitro* as described in the Materials and Methods. The reaction supernatants were subjected to immunoblot analysis using Glut-4 antibody (Panel-A), the immunopositive bands were quantitated by using Image-J program by computer analysis. The integrated area of each band was also calculated (Panel-B). Panel-A: Immunopositive bands of Glut-4 synthesis in GLS incubated in the presence of different concentrations of glucose in the incubation mixture as indicated. Panel-B: Integrated area of each of the immunopositive band as shown in the panel-A. Both Panel-A and Panel-B are the representative of experiments by using 6 different animals.

To determine whether the actual synthesis of Glut-4 occurred in the GLS was due to the increased synthesis of NO, or merely due to the release of preformed Glut-4 from the cells by NO, in parallel experiments the synthesis of Glut-4 in the GLS in the presence of glucose was determined by *in vitro* translation of Glut-4 mRNA as described in the Materials and Methods, and the quantitation of Glut-4 was made by immunoblot analysis. It was found that the integrated area of the Glut-4 band in the immunoblot in the absence of added glucose in the incubation mixture was 0.399 mm^2^ which was increased to 1.285±0.064 mm^2^ in the presence of 0.02M glucose in the incubation mixture (n=6, p<0.05). In contrast, increase of the glucose concentration from 0.02M to 0.03M in the incubation mixture actually resulted in the decreased synthesis of Glut-4, as estimated by the integrated area of the band which was 0.694±0.034mm^2^ (Figure 6). (n=6, p<0.05)

### The role of glucose induced NO synthesis in the expression of proinsulin genes I and II in the mice liver cells

**Figure 7:**
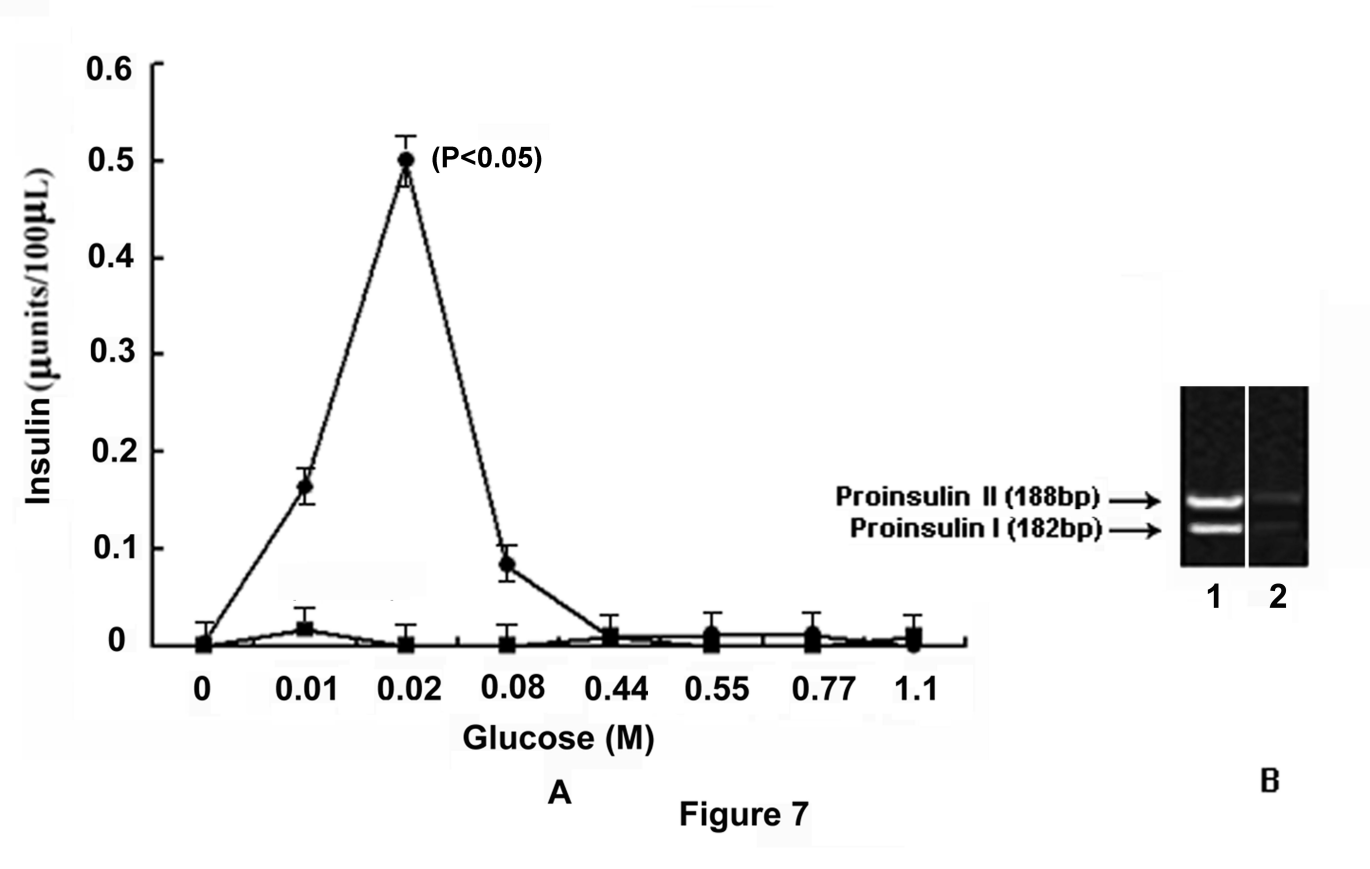
Role of NO in insulin synthesis in glucose treated GLS via the expression of proinsulin genes. The grated liver suspension in HEPES buffer, pH 7.4, was incubated with different amounts of glucose as indicated for 30 min at 37°C. After incubation, nucleic acids were extracted and insulin mRNA was translated *in* *vitro* as described in the Materials and Methods. The amount of insulin synthesized, was quantitated by ELISA using anti-insulin antibody. In parallel experiments, the incubation mixture was incubated with glucose and 0.1mM NAME and the synthesis of insulin was similarly determined. Panel-A: Glucose induced synthesis of insulin in the presence and absence of NAME. The solid circles (●) represent insulin synthesis in the presence of different amounts of glucose and the solid squares (■) represent the synthesis of insulin in the presence of both glucose and 0.1mM NAME. Panel-B: Agarose gel electrophoresis of cDNA prepared from the isolated insulin mRNA. ‘**1**’ and ‘**2**’ represents the expression of proinsulin genes I and II in the presence and absence of 0.02M glucose respectively. Results shown are representatives of the optical densities obtained from 6 different experiments using 6 different animals.

As glucose has been reported to have an essential role in the synthesis and secretion of insulin both in the pancreatic β cells [1,2] and in the adult mice hepatocytes [3], the role of glucose induced NO synthesis by GANOS in the liver cells was investigated to determine the role of NO, if any, in the actual synthesis of insulin in the mice liver cells in the presence of glucose. It was found that the treatment of GLS with different amounts of glucose resulted in the synthesis of insulin that was quantitated by ELISA [22]. It was found that at 0.02M glucose, the synthesis of insulin was maximally stimulated (0.5±0.025 µunit of insulin) over the control (0 µunit of insulin) (n=6, p< 0.05) (Figure 7A). The increased synthesis of insulin in the presence of glucose was related to the increase of glucose induced NO synthesis in the hepatocytes as described above (Figure 1). Addition of 0.1mM L-NAME to the reaction mixture was found to result in the inhibition of both insulin and NO syntheses at all concentrations of glucose used in the reaction mixture. The incubation of GLS with glucose showed the expression of both proinsulin genes I and II as determined by cDNA analysis (Figure 7B) which was expressed due to NO synthesis compared to control.

## Discussion

These results demonstrated that glucose which has an essential role in the synthesis and secretion of insulin both in the pancreatic β cells [1,2] and in the hepatocytes in the adult mice liver [3] had also a critically important of role both in the synthesis (Figure 5) and in the translocation of Glut-4 (Figure 4) for its own transportation from the external medium into the liver cells even in the presence of opposing effect of Glut-2 which is known to efflux the sugar from the liver cells into the circulation [13]. The effects of glucose on the synthesis and translocation of Glut-4 was found to be related to the stimulation of a constitutive form of NOS by glucose itself in the liver cells membranes. The existence of the glucose activated NOS in the liver cells, that could be critically important in the hepatic insulin synthesis has never been realized before. We however reported before the glucose induced NO synthesis was also involved in the glucose transportation in the islets of Langerhans for the synthesis and secretion of the hypoglycaemic protein [12]. As described in the Figure-5 the glucose induced increase of Glut-4 synthesis was maximally stimulated at 0.02M glucose and the increase of glucose concentration greater than 0.02M in the reaction mixture actually resulted in the reduction of Glut-4 synthesis in the liver cells. It was also noted that either 0.02M glucose or the use of NO itself instead of glucose was found to stimulate the Glut-4 synthesis in the reaction mixture. Furthermore the addition of 0.1mM NAME to the reaction mixture containing 0.02M glucose inhibited both NO and Glut-4 synthesis. The glucose induced synthesis of NO in the liver cell membrane was found to stimulate the actual synthesis of Glut-4 in the liver cells as demonstrated by the *in vitro* translation of mRNA of Glut-4 (Figure 6) and not merely due to the release of preformed Glut-4 from the liver cells by NO. 

However, the role of glucose induced NO synthesis by GANOS in the hepatocytes was not restricted only to the translocation and to the synthesis of Glut-4 in the liver cells, but the GANOS induced NO synthesis resulted in the stimulation of glucose induced insulin synthesis in these cells. Furthermore the glucose induced inhibition of NO synthesis in the liver cells by NAME was also found to result in the inhibition of the hormone synthesis even in the presence of glucose (Figure 7A). In other words, the glucose induced insulin synthesis in the liver cells cannot take place in the presence of glucose alone, as it is currently thought, but the simultaneous presence of NO along with glucose was apparently essential for the synthesis of insulin at least in the mice hepatocytes (Figure 7 A,B).

In the context of the role of NO in the hepatic insulin synthesis as described here, it should also be mentioned here that NO, is also reported to play both an essential role in the glucose induced synthesis and secretion of the hypoglycemic hormone in the pancreatic β cells [12]. Not only the oxide could stimulate uptake of glucose by the pancreatic cells, but NO was found to be involved both in the synthesis and secretion of insulin in the pancreatic β cells. Furthermore, estriol (an estrogen) and progesterone has been reported to stimulate insulin synthesis in the liver cells through NO synthesis [27]. These results suggested that glucose induced insulin synthesis was not an unique event produced by glucose induced NO synthesis, and the synthesis of insulin in the presence of glucose would also take place even in the synthesis of NO by estrogen and progesterone [27]. The role of NO in the synthesis of hepatic insulin was probably more important than in the pancreatic cells [3], in that, it has been claimed before that due to the absence of convertases and carboxy peptidases in the liver cells the proinsulin genes products in the liver cells could not be converted to bioactive insulin [28]. However we have reported before by 3 different lines of evidence, (I) that the insulin produced in the liver was found to be bioactive insulin as determined by bioassay using diabetic mice and by (II) Enzyme linked immunosorbent assay (ELISA) by using antibody from commercial source, (III) That the liver insulin was indeed insulin was confirmed by amino acid sequence analysis [3]. In the context that the hepatic cells were capable of producing bioactive insulin, we [29] and several other investigators [30–32] have reported before that different serine proteinases instead of carboxy peptidases and convertases were capable of converting the proinsulin gene products to bioactive insulin in the liver cells. As NO is reported to directly activate plasminogen in the circulation to plasmin in the absence of cells or cofactors [16], the glucose induced synthesis of NO through the stimulation of GANOS in the liver membrane, would convert plasminogen to plasmin (a serine proteinase) in the circulation which was reported before to be involved in the production of insulin in the liver cells [3].

However the effects of glucose induced NO synthesis as described above was not an open ended (i.e. non equilibrating) metabolic event. It has been reported that the process is under the regulatory control of a stress induced physiologic inhibitor of NO synthesis, identified to be dermcidin isoform 2, which is abundantly synthesized in hepatocytes, endothelial, muscle cells (unpublished) and in leucocytes [33]. This protein is found to be a potent inhibitor of all known forms of nitric oxide synthases [34] including GANOS (unpublished).

## Conclusion

It can be concluded from the results as described above on the glucose induced NO synthesis that the activation of GANOS in the liver cell membrane would play a critically important role both in the transportation of glucose and in the synthesis of bioactive insulin from proinsulin genes products in the liver cells. The presence of glucose alone in the liver cells was not sufficient by itself for the synthesis of insulin, and the activation of the liver membrane nitric oxide synthase by glucose played an essential role in the glucose induced synthesis of insulin, at least in the mice hepatocytes. 
